# Evaluating the feasibility of implementing a Telesleep pilot program using two-tiered external facilitation

**DOI:** 10.1186/s12913-020-05164-y

**Published:** 2020-04-26

**Authors:** Nicholas A. Rattray, Andrew Khaw, Mackenzie McGrath, Teresa M. Damush, Edward J. Miech, Adam Lenet, Stephanie Stahl, Jared Ferguson, Jennifer Myers, David Guenther, Barbara J. Homoya, Dawn M. Bravata

**Affiliations:** 1 VA Health Services Research & Development Center for Health Information and Communication (CHIC), Veterans Health Indiana, Roudebush VAMC, Indianapolis, Indiana USA; 2 William M. Tierney Center for Health Services Research, Regenstrief Institute, Inc., Indianapolis, Indiana USA; 3 VA Precision Monitoring (PRIS-M) QUERI, Veterans Health Indiana, Indianapolis, Indiana USA; 4 Department of Anthropology, Indiana University-Purdue University, Indianapolis, Indiana USA; 5 Indiana University School of Medicine, Indianapolis, Indiana USA; 6 Internal Medicine and Sleep Services, Veterans Health Indiana, Indianapolis, IN USA; 7 Department of Neurology, Indiana University School of Medicine, Indianapolis, IN USA; 8 Nursing Service, Veterans Health Indiana, Indianapolis, IN USA; 9 Department of Internal Medicine and Geriatrics, Indiana University School of Medicine, Indianapolis, Indiana USA

**Keywords:** Disease management, Sleep apnea, Outcomes, Telehealth, Sleep medicine, Quality improvement, Implementation science

## Abstract

**Background:**

Obstructive sleep apnea (OSA) can negatively impact patients’ health status and outcomes. Positive airway pressure (PAP) reverses airway obstruction and may reduce the risk of adverse outcomes. Remote monitoring of PAP (as opposed to in-person visits) may improve access to sleep medicine services. This study aimed to evaluate the feasibility of implementing a clinical program that delivers treatment for OSA through PAP remote monitoring using external facilitation as an implementation strategy.

**Methods:**

Participants included patients with OSA at a Veteran Affairs Medical Center (VAMC). PAP adherence and clinical disease severity on treatment (measured by the apnea hypopnea index [AHI]) were the preliminary effectiveness outcomes across two delivery models: usual care (in-person) and Telehealth nurse-delivered remote monitoring. We also assessed visit duration and travel distance. A prospective, mixed-methods evaluation examined the two-tiered external facilitation implementation strategy.

**Results:**

The pilot project included *N* = 52 usual care patients and *N* = 38 Telehealth nurse-delivered remote monitoring patients. PAP adherence and disease severity were similar across the delivery modalities. However, remote monitoring visits were 50% shorter than in-person visits and saved a mean of 72 miles of travel (median = 45.6, SD = 59.0, mode = 17.8, range 5.4–220). A total of 62 interviews were conducted during implementation with a purposive sample of 12 clinical staff involved in program implementation. Weekly external facilitation delivered to both front-line staff and supervisory physicians was necessary to ensure patient enrollment and treatment. Synchronized, “two-tiered” facilitation at the executive and coordinator levels proved crucial to developing the clinical and administrative infrastructure to support a PAP remote monitoring program and to overcome implementation barriers.

**Conclusions:**

Remote PAP monitoring had similar efficacy to in-person PAP services in this Veteran population. Although external facilitation is a widely-recognized implementation strategy in quality improvement projects, less is known about how multiple facilitators work together to help implement complex programs. Two–tiered facilitation offers a model well-suited to programs where innovations span disciplines, disrupt professional hierarchies (such as those between service chiefs, clinicians, and technicians) and bring together providers who do not know each other, yet must collaborate to improve access to care.

## Background

Sleep apnea is a common condition among Veterans, affecting 16–36% of Veterans [1]. An estimated 1.3 million Veterans with obstructive sleep apnea (OSA) are enrolled in Veterans Affairs (VA) health care. The diagnosis of OSA is made on the basis of the apnea hypopnea index (AHI) which describes the number of respiratory events observed during sleep; apneas are complete cessations in air flow and hypopneas are reductions in air flow. OSA is present if at least five respiratory events are observed per hour of sleep (i.e., AHI is ≥5 events/hour) [2]. Studies have demonstrated that untreated OSA can lead to increased healthcare cost and as well as negative consequences for patients [3]. Increased awareness of sleep apnea among Veterans may be due to changes in benefits. According to VA records, Veteran claims for service-connected benefits for sleep apnea increased nearly 150% between 2009 and 2014 after sleep apnea was recognized as a service-connected condition. Between 2005 and 2014, the prevalence of OSA has doubled among VA and military personnel [1]. Positive airway pressure (PAP) therapy helps maintain airways during sleep and is the most commonly used treatment for OSA [4, 5]. While VA provides an estimated 100,000 new PAP devices annually [6], ample room for improvement remains. Only 1 in 5 VA patients with sleep apnea are cared for by VA sleep medicine service; of that population, 45% use PAP; and only about half of those patients have effective therapy (e.g., no leak) [7]. Providing diagnostic and treatment services for this large and growing population has strained existing VA sleep infrastructure and access [8], which itself varies widely across medical centers [9]. VA has addressed OSA through multiple modalities that include Telehealth and clinic-based treatment. Compared to in-person clinic treatment, remote monitoring of home-based PAP machines has been shown to be comparable in terms of clinical outcomes and patient satisfaction [10–12].

Patient access challenges with PAP in the VA are multiple. Although many veterans receive PAP machines with remote monitoring capability, few VAMCs currently use this feature. Hesitance to adopt remote PAP monitoring may stem in part from a concern that enabling remote PAP monitoring could generate a new influx of data from patients’ homes to the medical center for which no facility-level clinical infrastructure exists for ongoing monitoring and follow-up as needed. Furthermore, caring for patients remotely moves beyond the current “patient-comes-to-the-facility” framework of providing in-person service to individual patients at sleep clinics towards a “facility-comes-to-the-patient” model where care primarily takes place in patient homes [8]. In the current in-person clinic model, VA patients must travel to medical facilities for appointments with sleep medicine services even if only to download their PAP data, but wait times for PAP/sleep clinics are among the longest of any clinic at many VAMCs.

Upon request by the VA Medical Director to increase patient access, we implemented a quality improvement (QI) project at the Indianapolis VAMC to pilot a Telesleep model, taking advantage of remote PAP monitoring to deliver high quality, accessible care to Veterans with OSA. The strategic goals of the project were to develop clinical algorithms and workflow for remote PAP monitoring, to implement a Telesleep program aimed at improving clinical outcomes for Veterans with obstructive sleep apnea (e.g., PAP use), to improve access to sleep clinics, and to enable patients to engage in OSA self-management via access to their own PAP data. Using a prospective, mixed methods design [13], we evaluated the implementation of the Telesleep quality improvement project that involved remote PAP monitoring by Telehealth nurses with two primary aims. First, we sought to compare Telesleep patients to usual care (in-person) patients. We hypothesized that compared to usual care patients, Telesleep patients would have similar OSA outcomes measured at 180 days, acceptable patient satisfaction, and reduced patient travel costs. Our second aim was to assess the impact of the external facilitation implementation strategy on acceptability by providers and in addressing barriers to increase adoption. We hypothesized that external facilitation could identify solutions to key implementation barriers. We report results drawing on the Medical Research Council (MRC) Framework for complex interventions [14].

## Methods

### Setting

This pragmatic feasibility pilot study was conducted at a tertiary VAMC located in Indianapolis, IN and affiliated with the Indiana University School of Medicine. Of the 60,000 Veteran patients treated annually, approximately 13,000 (21.7%) have a sleep apnea diagnosis. In federal fiscal year (FY) 2017, the sleep medicine service had 13,678 encounters for 6808 unique patients; it was staffed by two physicians and seven PAP respiratory therapists. Approximately 1800 new patients are diagnosed with sleep apnea each year at this VA facility. Usual treatment of patients with OSA involved consultation with sleep medicine physicians and in-person visits with respiratory technicians. The Telehealth service at this same facility has been primarily staffed by nurse case managers who work with patients who have chronic conditions (e.g., chronic heart failure) by providing regular, individualized attention to Veterans and reducing the need for in-person visits [15]. Overall, 37.4% of patients served by this medical facility are classified as living in “highly rural” or “rural” settings.

We evaluated the implementation of a pilot quality improvement “Telesleep” program within the sleep and Telehealth services. The evaluation study was part of the Precision Monitoring to Transform Care (PRIS-M) QUERI Center, which has approval from the Indiana University Institutional Review Board and the Roudebush VAMC Research and Development Committee. A secondary analysis of deidentified data collected in the QI project was performed.

### Intervention: Telesleep quality improvement program

**Fig. 1:**
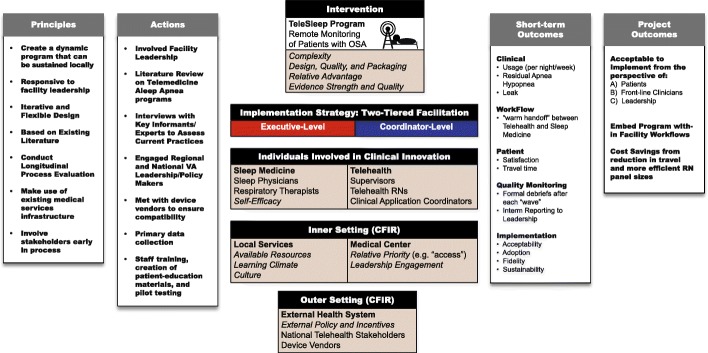
Logic Model for Intervention Development

The Telesleep clinical pathway is described in Additional file 1. Telesleep was a quality improvement program, and as such, did not have a published protocol. Figure 1 depicts a logic model that guided development of the quality improvement program. The PAP devices that were used for remote monitoring were ResMed AirSense-10 PAP machines with wireless capability, issued by VA prosthetics. Sleep service staff performed the initial setup of the device and helped the patient with mask fitting and education at the VAMC. After a patient handoff by the sleep technician, the Telehealth service was responsible for patient follow-up via telephone. Data about PAP adherence and residual AHI were obtained from the remote monitoring PAP portal (AirView, ResMed) for all patients newly initiated on PAP therapy. The Telehealth nurses used the clinical pathway (Additional file 1) to guide their responses to data the observed in the web-based portal (e.g., if there no PAP use, they inquired about barriers to use and provided education and support).

### Clinical and patient-centered measures

#### Clinical effectiveness

Clinical effectiveness was measured in terms of adherence (hours per night and percentage of nights used) and clinical disease severity measured in terms of the residual apnea hypopnea index (AHI in events per hour). PAP adherence is routinely assessed against the standard of > 4 h/night for > 70% of nights in clinical studies, which is the threshold used for authorized non-VA reimbursement for PAP [16–21]. The AHI is an accepted method for diagnosis OSA and is also a key metric of disease severity [22, 23]. PAP adherence in median hours of use per night and the residual AHI were measured directly by the PAP machine and assessed at 30-days, 90-days, and 180-days after PAP initiation. The primary assessment of clinical effectiveness was made on the basis of the 180-day clinical effectiveness outcomes. Clinical effectiveness was defined as the proportion of patients with excellent adherence, defined as ≥4 h/night for > 70% of nights. Clinical disease control was considered excellent if the AHI was < 5 events per hour.

#### Patient-centered outcomes

Two primary patient-centered outcomes were evaluated: patient satisfaction and Veteran travel distance. Patient satisfaction was assessed utilizing an Automated Voice Response system (AVR) in which patients were contacted by telephone and asked their opinion regarding their experience with the overall care they received at the local VAMC as well as the specific care they received related to the treatment of their OSA. To minimize bias, all Veterans meeting the specified inclusion criteria with an outpatient appointment with specified sleep technicians were approached and invited to participate in the Telesleep program. All enrolled veterans were asked to use the ATR using the same methods at the same point of time within the program. All enrollees had access to a telephone. Calls went to both usual care and the Telesleep participants. Patients were asked to provide their response to the questions based on a scale from zero to nine, with zero being very unsatisfied and nine representing very satisfied. In this analysis, an answer of eight or higher was considered “satisfied.” The economic evaluation focused on beneficiary travel pay because this was a key metric identified by the facility leadership. The mileage reimbursement rate for the VA is 41.5 cents per mile. The distance from the Veteran’s primary residence to the VA facility was calculated for each Veteran in the Telesleep and control groups. As a secondary measure, we also asked sleep technicians to estimate the number of minutes spent in an in-person visit or in the telephone-based remote monitoring visit (include time for repeat telephone calls and documentation).

### Analyses

#### Evaluation of clinical and patient centered outcomes

For the evaluation of the clinical effectiveness outcomes, we compared new OSA patients enrolled in the pilot Telesleep program (February to June 2016) with those receiving usual care (on-site clinic visits for follow-up care). We focused on new patients to limit bias because many existing patients would have sufficient Continuous Positive Airway Pressure (CPAP) adherence; thus, although existing patients were offered Telesleep, our analysis only included new patients. All new patients who received CPAP machines were eligible with no exclusion criteria. Because this was a quality improvement project aimed at assessing feasibility, the study size was not pre-calculated, but rather was constrained by a typical Telehealth nurse panel, which is 90 patients. The telehealth service of the targeted hospital agreed to designate two of its credentialed telehealth nurses to the Telesleep program. For the evaluation of the patient centered outcomes, all patients in the Telesleep program were compared with usual care control patients. Descriptive statistics were calculated for driving time and patient satisfaction surveys. Rates were reported and comparisons in rates were made using the Fisher exact test for categorical variables and t-tests for continuous variables; two-sided *p*-values were reported.

#### Implementation evaluation

We used the Consolidated Framework for Implementation Research (CFIR), a theory- and evidence-based typology for understanding implementation and hypothesizing mechanisms of change in a health services context, to guide our evaluation [24]. The definition of facilitation in the present study draws from recent efforts to clarify its role in health care settings. In the CFIR “Process” domain, “external change agents” are those who are outside of an entity who formally facilitate intervention decisions in a desirable direction. Our “two-tiered” facilitators were external agents working at different levels within the organization.

To evaluate implementation of the program, we recruited a purposive sample of providers from the Sleep and Telehealth services. Participants included 12 staff members, including three physicians, five Telehealth nurses, and two respiratory therapists. The evaluation team monitored the program delivery weekly through the reviews of the telehealth nurse delivery spreadsheet; group meetings with the facilitators, telehealth nurses and service chiefs to understand barriers and facilitators; and through bimonthly individual brief interviews with the clinical providers. In terms of fidelity/adaptation, the Telesleep program had core clinical elements that needed to be delivered with fidelity while other more peripheral elements were permitted to be locally adapted. Participants agreed to take part in brief check-ins approximately twice a month throughout the active implementation period; no exclusion criteria applied. Individual interviews consisted of brief (5–10 min) check-in sessions aimed at eliciting participants’ perception of implementation at a given point in time. These sessions took place every two to 3 weeks as an in-person or phone conversation. Participants responded to a variant of the question, “What are some things that have happened over the past 2-3 weeks that, from your perspective, are relevant to implementing the project”? Each discrete activity was termed “an update,” and was verbally scored from a range from of + 3 to − 3 that indicated the type of impact and its influence (+ 3 = strong positive, + 2 = moderate positive, + 1 = weak positive, 0 = neutral, − 1 = weak negative, − 2 = moderate negative, − 3 = strong negative). More details on the methodological approach used in this study are available elsewhere [25]. Table 1 illustrates this technique with four exemplar updates. Five members of the research team conducted sessions with 12 different staff members over the course of a six-month period; 62 sessions were conducted, resulting in 190 updates. Data was fed back regularly to the facilitators.

**Table 1:** Example of the 4-column template populated with updates, ratings, and comments

Update	Score	Rationale	Comments
Telehealth nurse shadowed a sleep physician	+ 1	Successful experience for nurses, who observed while sleep doc set up a new patient with PAP^a^ in Sleep Clinic	Reported that small events like this help people start to think about the notion of “Telesleep”
Telesleep recruitment continues to be “slow”	−2	In wave 1, there were no “warm-hand offs” from Respiratory to Telehealth services following the protocol.	The participant suggested that inertia was at play here, and that PAP technicians are somewhat resistant. All patients had to be called by Telehealth nurses
New PAP therapist is hired	+ 2	The new PAP therapist is currently working in sleep medicine and is being trained on the ResMed PAP machines.	This participant anticipates that having a new therapist will boost enrollment in the pilot.
New note template in electronic health record system was confusing	−2	Telehealth nurses found the wording on the note to be confusing as did the sleep service and claimed this affected implementation.	Although the note was initially confusing, it was revised with input until all users were satisfied. Future updates may be more positive.

^a^PAP refers to positive airway pressure

Two PhD-level researchers used NVivo 11 (QSR International, Australia) to manage and code qualitative data. Each person who conducted ongoing check-ins on implementation progress with staff composed analytic memos summarizing clinicians’ acceptability of the program. Team members met to share and discuss preliminary findings, and to arrive at consensus regarding key findings.

## Results

### Patient demographics

**Fig. 2:**
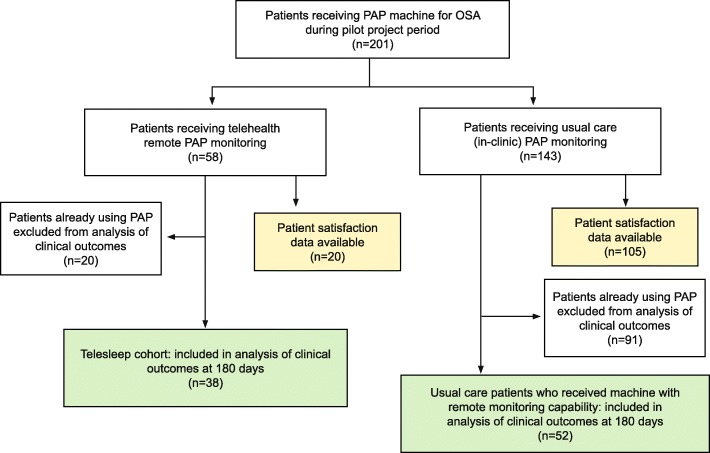
Flow of Patients and Measurements

The Telesleep program was pilot-tested with 38 patients who were compared with 52 usual care patients who were new PAP users. The baseline characteristics of the two groups were statistically similar (Table 2). Figure 2 describes measurement points in the clinical quality improvement program.

**Table 2:** Baseline characteristics of control and intervention groups

Characteristic	Telesleep Remote Monitoring *N* = 38	Usual Care Control *N* = 52	***P***-value
Age (years): mean ± standard deviation (range)	54.9 ± 13.9 (28.5–73.7)	56.2 ± 15.5 (26.7–81.1)	0.683
Male Gender: % (n)	89.5% (34)	96.2% (50)	0.236
Race: % (n)	–	–	1.000
White	86.8% (33)	84.6% (44)	–
Black	10.5% (4)	15.4% (8)	–
Asian	0% (0)	0% (0)	–
Other	0% (0)	0% (0)	–
Unknown	2.6% (1)	0% (0)	–
Hispanic ethnicity: % (n)	0% (0)	2.6% (1)	1.000
Hypertension: % (n)	65.8% (25)	69.2% (36)	0.821
Chronic obstructive pulmonary Disease (COPD): % (n)	2.6% (1)	11.5% (6)	0.231
Stroke: % (n)	2.6% (1)	7.7% (4)	0.392
Cognitive Impairment: % (n)	0.0% (0)	3.9% (2)	0.507
Opioids present: % (n)	15.8% (6)	25.0% (13)	0.433
Baseline apnea hypopnea index (AHI) (events/h): Mean ± standard deviation (range)	22.1 ± 19.8 (5.0–84.6)	26.2 ± 22.7 (5.5–89.8)	0.375

### Clinical effectiveness

Table 3 indicates that program participants had similar PAP adherence over the course of the 180-day data collection period (32% for Telesleep versus 23% for usual care, *p*-value = 0.470), and similar disease control (86.8% of Telesleep patients had a residual AHI of < 5 events/hour, versus 82.7% for usual care, p-value = 0.770). Table 3 also indicates that the adherence and disease control measures remained relatively stable over the three assessment periods: 30-days, 90-days and 180-days. For example, the median hours of PAP use per night among intervention patients across the three assessment periods was: 4.6 ± 2.5, 4.6 ± 2.4, and 4.6 ± 2.5. Similarly, the median hours of PAP use per night among usual care patients was: 4.2 ± 2.8, 4.1 ± 2.6, and 4.0 ± 2.7.

**Table 3:** Clinical Effectiveness

Clinical Outcome Measures	Telesleep Remote Monitoring *N* = 38	Usual Care Control *N* = 52	*P*-value
**30-Day Measures**			
Median Hours Used per Night (hours): Mean ± standard deviation (range)	4.6 ± 2.5 (0.02–8.8)	4.2 ± 2.8 (0.0–9.4)	0.486
Residual AHI (events/h):^a^ Mean ± standard deviation (range)	3.2 ± 3.8 (0.0–18.1)	4.4 ± 7.1 (0.0–35.2)	0.347
Residual AHI < 5 events/h: % (n)	81.6% (31)	82.7% (43)	1.000
**90-Day Measures**			
Median Hours Used per Night (hours): Mean ± standard deviation (range)	4.6 ± 2.4 (0.02–8.9)	4.1 ± 2.6 (0.0–8.6)	0.355
Residual AHI (events/h): Mean (range)	2.8 ± 2.6 (0.0–10.3)	3.6 ± 4.9 (0.0–30.3)	0.362
Residual AHI < 5 events/h: % (n)	81.6% (31)	84.6% (44)	0.778
**180-Day Measures**			
Median Hours Used per Night (hours): Mean ± standard deviation (range)	4.6 ± 2.5 (0.06–9.07)	4.0 ± 2.7 (0.0–9.13)	0.286
Residual AHI (events/h): Mean ± standard deviation (range)	2.6 ± 2.5 (0.0–10.1)	3.6 ± 5.4 (0.0–34.3)	0.292
Residual AHI < 5 events/h: % (n)	86.8% (33)	82.7% (43)	0.770
Adherent (≥4 h/night for > 70% of nights)	32% (12)	23% (12)	0.470
Non-Adherent (< 4 h/night for < 70% of nights)	68% (26)	77% (40)	
Adherent and disease control (Residual AHI < 5 events/h)	32% (12)	21% (11)	0.330

^a^Residual AHI refers to the apnea hypopnea index recorded by the positive airway pressure (PAP) machine and denotes the degree to which the PAP eliminates respiratory events during sleep

### Patient centered outcomes

Patients in both the Telesleep and usual care groups were generally highly satisfied with their care (Table 4). In response to the AVR questions, “How satisfied are you with the care you have received for your sleep apnea?” 75.0% of respondents in the Telesleep program indicated that they were “mostly” [8] or “very” [9] satisfied, versus 64.8% in the usual care group (*p*-value = 0.447). Among the Telesleep program participants, 5.0% report “mostly” or “very” unsatisfied, versus 5.7% in the usual care group (*p*-value = 1.000). For comparison purposes, when asked “How satisfied are you with the care you received at the [left intentionally blank] VA medical center?,” 65.0% of Telesleep participants were mostly or very satisfied versus 67.6% of usual care patients (*p*-value = 0.801).

**Table 4:** Patient Satisfaction

Response Categories	Telesleep Remote Monitoring *N* = 20	Usual Care Control *N* = 105
	% (n)	% (n)
“How satisfied are you with the care you have received for your sleep apnea?”		
Mostly-very satisfied	75.0% (15)	64.8% (68)
Minimally-somewhat satisfied	0.0% (0)	15.2% (16)
Neutral	20.0% (4)	11.4% (12)
Slightly-somewhat unsatisfied	0.0% (0)	2.9% (3)
Mostly-very unsatisfied	5.0% (1)	5.7% (6)
“How satisfied are you with the care you received at the [BLANK] VA medical center?”		
Mostly-very satisfied	65.0% (13)	67.6% (71)
Minimally-somewhat satisfied	10.0% (2)	16.2% (17)
Neutral	15.0% (3)	11.4% (12)
Minimally-somewhat unsatisfied	0.0% (0)	1.9% (2)
Mostly-very unsatisfied	10.0% (2)	2.9% (3)

Participants in the Telesleep program lived an average of 72.9 roundtrip-miles from the facility (range: 5.4 to 220). Given a mean 72.9 mile roundtrip would save the VA facility approximately $29.82 (range $2.24 to $91.3) per patient per avoided in-person clinic visit.

### Implementation

The implementation evaluation focused on four main domains: acceptability, enrollment, external facilitation, and sustainability.

#### Acceptability

Front-line staff in each of the two services described related, but distinct, perceptions about the acceptability of the Telesleep program. Telehealth nurses described professional satisfaction with receiving additional training to deal with a clinically important medical condition. PAP technicians expressed doubt about the appropriateness of the Telesleep program (as implemented using Telehealth), concerns with increased workload, and reluctance to train Telehealth nurses. Yet following the second critical juncture that occurred after the joint service lunch, and after issues with professional boundaries were clarified, they overcame their initial resistance to the program.

#### Enrollment

In early enrollment waves, adoption by PAP technicians and nurses was gradual. The PAP technicians were the gatekeepers for enrollment and generating the handoffs across the services. Initially, enrollment by PAP technicians trickled in and they provided no warm handoffs to Telehealth. The CF developed a paper hand-off sheet to direct the enrolled patient to the Telehealth service and a patient tracking tool. However, by the latter half of the project, the nurses were at full capacity and the PAP technicians had to halt patient enrollment due to staffing constraints.

#### External facilitation

Following planning and development in 2015, the pilot program was active between February and June 2016. In the pre-implementation phase, the executive-level external facilitator (EF) convened a series of 25 planning meetings with a range of stakeholders including the national office for Telehealth, regional VA leaders, and other VAMCs. These activities aligned with the “Cosmopolitan” construct in the CFIR framework [24], as they involved the EF voluntarily reaching out to entities external to the facility in ways that could help implement the Telesleep program locally. Additional meetings initiated by the EF included soliciting input from device vendors and engaging local facility leadership outside of the target services to align priorities and establish the scope and budget of the project. Working closely with the coordinator-level external facilitator (CF), the EF identified key stakeholders and front-line staff and sought to understand their motivation for participation during weekly planning meetings in the pre-implementation phase. The clinical pathway was developed with input from respective service chiefs (sleep medicine and Telehealth services). The CF attended 21 of the pre-implementation meetings with an emphasis on supporting the needs of front-line providers involved in Telesleep.

During the implementation phase, 15 weekly progress meetings were held that were attended by front-line staff and leadership from both services in addition to support staff involved in specific implementation tasks. The CF addressed barriers to communication and training in multiple ways: by providing coaching on clinical issues for Telehealth nurses; fixing problems with patient tracking tools; offering performance data feedback to front-line providers; and aiding with the warm-handoff between PAP technicians and Telehealth nurses. As the CF described, building rapport with both sets of providers was important: “They got to see me and recognize me as somebody that was teaching, that had this knowledge, that I was the point person to interact with.” In addition, the CF convened two separate meetings to address issues with cross-service communication. The CF played the role of a neutral helper who had both clinical and technical expertise.

As implementation proceeded, both the EF and the CF were involved in monitoring implementation progress and feeding back information to both executive-level stakeholders and front-line staff. One example of this teamwork was illustrated when the CF helped develop new electronic medical record templates to document Telesleep care:

“It was the team—-all of us, sleep service, Telehealth, [EF] and I —working together to devise what was going to be in a Telehealth note. I would take those ideas, put them into plain text documents, then we’d give to the [clinical application coordinators] CACs who then would integrate them into actual [medical record] notes.”

The above example shows how developing tools for proper documentation was accomplished by eliciting feedback from providers and then translating preferences into useful, working electronic medical record templates. Prompt, direct communication from the CF to those implementing the program was likewise critical. As the CF recalled:

“That’s what won my favor with the Telehealth nurses: here’s someone that you contact, and he fixes it right now … you don’t have to wait till next Friday.”

Both the EF and the CF offered prompt feedback to ensure that implementation progress was not impeded by cross-service barriers.

**Fig. 3:**
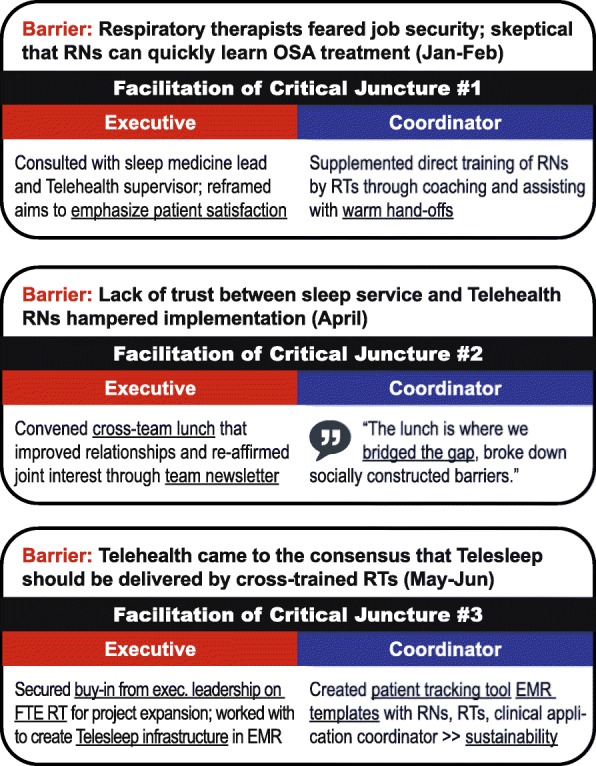
Three Critical Junctures in Implementation Process

At three key turning points the program was in danger of being discontinued. These can be seen as “critical junctures,” which refer to uncertain situations or moments where actions could lead to divergent pathways [26]. At each of these moments, deliberate actions at executive and coordinator levels enabled the project to proceed with enrollment. The role of the facilitators at each of these junctures is summarized in Fig. 3.

The first critical juncture occurred early in the active implementation phase as the initial patients were enrolled in February 2016. Whereas the goal for the first wave was 30 patients, only 5 patients had been enrolled at that point in time. Check-in sessions with sleep medicine staff revealed that PAP technicians were reluctant to train Telehealth nurses due to concerns about encroachment on their professional “turf.” In addition, they were reluctant to use new PAP machines and skeptical about the capacity of the nurses. Upon receiving this information, the facilitation team implemented a new approach to encouraging handoffs that also involved supplemental training of the nurses by the CF. One nurse emphasized how the CF was “so positive and helpful,” noting that adjustments to the clinical pathway made it easier to use.

Weekly sessions with front-line staff indicated that staff morale was an ongoing issue. While enrollment had increased, the EF and the CF received input that the process of bridging the two services into a Telesleep team had stalled, representing a second critical juncture. The EF and the CF scheduled a lunch event intended to bring the two services together, emphasizing how the program was aimed at improving Veterans’ health outcomes, with feedback on patient satisfaction to the program, and geared toward appreciation of providers. The lunch was held in early April and resulted in improved social relationships between PAP technicians and Telehealth nurses. During the lunch, the EF highlighted positive preliminary findings from the pilot, including testimonials from Veterans about reductions in their travel time. Feedback from the lunch suggested that PAP technicians and Telehealth nurses found common ground in improving patient care; following the lunch staff reported that they felt that they finally understood the Telesleep program.

The third critical juncture occurred in the final wave of patient enrollment. Following the lunch, patient enrollment was so successful that it had to be temporarily halted in May due to reaching full capacity in the Telehealth service. The CF created a patient tracking tool for nurses to manage the panel of Telesleep patients and worked closely with clinical applications coordinators to ensure that the templated electronic documentation was functional. The EF presented a business case assessment of the pilot study to facility leadership and outlined necessary infrastructure for expansion. Data gathered from front-line staff was instrumental in understanding a critical decision about whether the facility should implement a remote PAP monitoring program delivered through PAP technicians or by Telehealth nurses.

#### Sustainability

Following the pilot program, the EF presented data on clinical effectiveness, evidence of patient and staff satisfaction, and a business case analysis suggesting potential cost savings with the Telesleep program. As a result of the EF’s presentation, the facility leadership approved a Rapid Process Improvement Workshop in January 2017 [27]. The workshop led the facility to adopt the program by funding a new full-time PAP technician position dedicated to Telesleep treatment, which was also seen as a model program to be expanded at other VAMCs in the regional network.

## Discussion

Evaluation of this Telesleep quality improvement project revealed that remote monitoring was similar in terms of clinical effectiveness as the usual care approach which relied on in-person clinic visits. The program has the potential to improve access to care by reducing the time for patient visits. In addition, remote monitoring may reduce travel time for patients.

The observed rate of excellent PAP adherence was lower than we had expected but was similar to data reported from other VA populations. For example, in an evaluation of in-person versus telemedicine care, the proportion of nights patients used their PAP device ranged from 54 to 65% and the proportion of nights with at least 4 h of use ranged from 39 to 47% [8]. It may be that the observed rates of usage are lower than reported rates from populations where reimbursement policies remove PAP machines from patients with lower usage because the Department of Veterans Affairs does not remove PAP therapy from Veterans once the machines have been issued.

Implementation of remote PAP monitoring may be beneficial from a facility administrative perspective. Specifically, savings in beneficiary travel pay would be maximized by offering remote PAP monitoring to both new (N ~ 336) and existing (N ~ 2429) patients who are designated as living in rural areas (*N* = 2765) and hence have the longest travel times (an estimate of 138.6 miles per rural-dwelling patient). Given that the standard of care is to offer Veterans with a new PAP set-up a follow-up visit at 1 month post-set up and then visits annually, the implementation of a remote PAP monitoring program would save one visit per year (i.e., the 30-day follow-up visit) and the facility would save approximate $159,040 (2765 patients × 138.6 miles × 0.415 cents per mile) per year. In terms of access, Telehealth visits took half the time of the typical 40-min in-person visit (including all repeat phone calls and documentation), and therefore has the potential to improve sleep clinic access.

Evaluation of the implementation of this novel but complex program revealed underlying challenges. Because the program was predicated on “buy-in” from two separate sets of front-line staff, each service initially resisted moving beyond their typical scope of work. Specific barriers to implementation included significant staff training, new infrastructure, lack of rapport between front-line staff, and existing limitations in staff capacity. Under these conditions, the implementation strategy of external facilitation at the executive and coordinator levels—working both on their own and in concert—proved essential to implementation success. The two facilitators detected unexpected problems in the implementation process and proposed solutions that were palatable to project stakeholders, which others have characterized as “sensemaking.” [28] The two-tiered facilitation was designed by the external facilitators to overcome identified barriers and to honor preferences of front-line clinical staff. Because this complex program revealed unanticipated barriers, agile sensemaking on the part of the facilitators was necessary to help each unit reorganize their clinical practices.

This QI program entailed evaluating a complex intervention [14, 29, 30]. Two-tiered, external facilitation, which simultaneously included both executive and coordinator levels, supported the implementation of the Telesleep program at a single facility. This use of multiple facilitators for onsite implementation has previously been referred to in a different context as a “Partnered Facilitation Strategy.” [31] Similar in some ways to the two-tiered facilitation strategy described in this study, a “blended facilitator” within the partnered model represents two levels − a National External Implementation Facilitator expert and an Internal Regional Facilitator − and has been used in other quality improvement programs [32, 33].

This current evaluation offers insights into understanding how facilitation is both a role and a process [34]. Because the quality improvement project under study was non-mandated and involved developing a new program that bridged two services, a dynamic implementation strategy was critical to successful implementation. Working in tandem, both the EF and the CF focused not only on launching the program but also on increasing the capacity of the two different services to review performance data and evidence related to sleep apnea patients and respond accordingly. Specifically, the EF encouraged clinical managers to engage directly with front-line staff around metrics, quality improvement and professional development. Similarly, the CF supported both the PAP technicians and nurses in monitoring and adjusting the care they provided to Veterans. More broadly, facilitation can be conceptualized as a “meta-routine,” or bundles of activities that an organization draws upon to recognize and assimilate new knowledge. In the present study, two-tiered facilitation simultaneously addressed implementation barriers and fostered a robust capacity for learning and adaption in the new Telesleep program [35]. Within the program, staff were heterogeneous and it was unknown what their personal perspectives on the innovations were prior to implementation, which is a known barrier to adoption of new technologies in healthcare [36].

Several limitations are worth noting. First, the findings presented here are based solely on a single medical facility with a limited number of participants; this geographic context may represent a distinctive situation. Second, results obtained from veteran patients may not be generalizable to non-veteran populations given differences in gender (male predominance among veterans) and comorbidity (a greater overall comorbid burden and increased prevalence of certain comorbidities such as post-traumatic stress disorder among veterans). Given that OSA is more common in patients with post-traumatic stress disorder, traumatic brain injuries, and cardiovascular disease-conditions that are highly prevalent among Veterans, there is a strong possibility that OSA is under-diagnosed among Veterans [37–39]. Third, as a national system of government-funded health care, VA sleep medicine may differ from other health systems, especially those where PAP management is guided by health insurance directives. However, remote PAP monitoring has been increasing in non-VA settings [40, 41]. Fourth, participants were not randomly allocated to the two groups. Fifth, only one manufacturer’s CPAP machine was used in this study. However, patients in both the remote and in-person monitoring groups used the same machine type, therefore, it is unlikely that the machine itself caused differences in the two groups. Also, given that most CPAP manufacturers offer remote monitoring capability, it is unlikely that results would differ across manufacturers. Sixth, the program used a clinical pathway rather than a research protocol because it was a quality improvement project. Seventh, a formal economic evaluation was out of scope of this project. Finally, although respondent bias in the interview data collected prospectively was a concern, the frequency and duration of participant interviews was a strength of the study. Future efforts may address how to deliver seamless and integrated traditional and remote sleep medicine within a healthcare system that serve community-based, non-veteran populations.

## Conclusion

The results indicating that a Telesleep pilot project was associated with high patient satisfaction, comparable outcomes on clinical sleep apnea management measures, and economic data demonstrating potential costs savings favor the implementation of remote PAP monitoring. In applying implementation science to the evaluation of a novel pilot program, this study contributes to understanding of how external facilitation works as an implementation strategy. Two-tiered facilitation may provide a useful model of external facilitation when innovations span disciplines and disturb professional hierarchies such as those between chiefs, clinicians, and technicians.

## Supplementary information

**Additional file 1.** Remote PAP Telesleep Quality Improvement program protocol.

## Data Availability

To safeguard the privacy of participating staff, the dataset used in the current study is only available in a de-identified version from the corresponding author upon reasonable request.

## Abbreviations

AHI: Apnea hypopnea index
AVR: Automated voice response system
CHIC: Center for health information and communication
CF: Coordinator-level external facilitator
CFIR: Consolidated framework for implementation research
CPAP: Continuous positive airway pressure
EF: Executive-level external facilitator
MRC: Medical research council
OSA: Obstructive sleep apnea
PAP: Positive airway pressure
QUERI: Quality enhanced research initiative
QI: Quality improvement
PRIS-M: Va precision monitoring
VA: Veterans affairs
VAMC: Veteran affairs medical center
