# Supplementary material for: Evaluating the feasibility of implementing a Telesleep pilot program using two-tiered external facilitation
Source: BMC Health Serv Res. 2020 Apr 26;20:357. doi: 10.1186/s12913-020-05164-y (PMC7183618; doi:10.1186/s12913-020-05164-y)
Supplement: Supplementary file 1 — Additional file 1. Remote PAP Telesleep Quality Improvement program protocol. [file 12913_2020_5164_MOESM1_ESM.pdf]

REMOTE PAP PROGRAM PROTOCOL

MEDIAN LEAK (L/min)	RESIDUAL AHI		ADHERENCE		ACTION	FOLLOW-UP
			Hours per night	% of nights used		
≥24	Any		Any	Any	Address leak first (see below)	See Below
<24	>5	[(HI + Obstructive AHI)/Total AHI] x 100 ≥ 50%			Increase pressure by 2 cm H_2O per CPAP<br>OR<br>BiPAP Increase pressure by 2 cm H_2O on both levels	One Week
	>5	[(HI + Obstructive AHI)/Total AHI] x 100 < 50%			Sleep MD notification on note	Sleep MD will determine follow-up but will not be discharged from TeleHealth unless MD notifies TeleHealth Svcs
-	-	-	No use		Inquire about barriers to use, provide education	3 attempted contacts, if no success in reaching patient, then PAP clinic consult
-	Any		<1 hour per night		Inquire about barriers to use	Weekly basis for 3 weeks after day 7 and then referred to the sleep MD for PAP noncompliance if remains noncompliant after the 4th week
			<4	>70%	Encourage use whenever patient is sleeping	-
			>4	<70%	Ask about days without use	Weekly basis for 3 weeks after day 7 and then referred to the sleep MD for PAP noncompliance if remains noncompliant after the 4th week

PATIENT PROBLEM		ACTION	FOLLOW-UP
Mask	Air leaking around mask, new mask	PAP clinic for mask re-fitting	Follow up at Routine Time
	Air leaking around mask, mask ≥4 weeks old and leak was not previously a problem	Prosthetics consult: new cushion	2 Weeks
	Need to tighten mask	Prosthetics consult: new cushion	2 Weeks
	Other poor mask fit issues	PAP clinic for mask re-fitting	Follow up at Routine Time
	Claustrophobia or trouble getting used to mask	Encourage to use during awake time to get more adapted to PAP	One week

Version20160509

REMOTE PAP PROGRAM PROTOCOL

Leak	Sleeps with mouth open	Ask about nasal congestion (see below). Order chin strap if nasal congestion not a major contributor.	One week, if no improvement schedule PAP clinic appointment
	Poor mask fit (see mask issues above)		
Adherence	Poor adherence, not motivated to use PAP	Inquire about barriers to use, provide education and encouragement	
Other	Dry mouth	Adjust humidity level. Ask about sleeping with mouth open (see above). If both ineffective, then can try an OTC mouthwash, spray, or toothpaste for dry mouth (e.g., Biotene).	2 Weeks
	Pressure intolerance	Use ramp. If ineffective, use during awake time while relaxed (e.g., while watching TV) to become adapted to PAP	1 Week
	Claustrophobia	Encourage use during awake time while relaxed (as above)	1 Week
	Nasal dryness	Adjust humidity level. If ineffective, place an order for nasal saline spray for PCP to co-sign or patient can get nasal saline gel or nasal saline spray OTC	Routine Follow-Up
	Nasal congestion	Adjust humidity level. If ineffective, try an OTC nasal saline rinse	Routine Follow-Up
	Snoring	Leak / Adherence is outside normal limits	Follow protocol for Leak & Adherence
		Leak / Adherence is with normal limits	Notify patient that airway inflammation may decrease with continued PAP use resulting in snore decrease with continued PAP use.
	Condensation in tubing or mask	Options include: Reduce humidity setting, lower CPAP unit to a level below the bed, insulate tubing (can use ace bandage, tube socks, pre-made tubing wraps)	Routine Follow-Up

CONTACTS	
**ISSUE**	**CONTACT**
Scheduling (e.g., patient needs to be seen in PAP clinic (RTs) or Sleep Medicine Clinic (MDs))	
Clinical issues (e.g., questions about what to do about an elevated AHI)	
Mask, supply, or machine related issues	
ResMed Technical Help	

Version20160509
